# Outcomes of Klebsiella endogenous endophthalmitis treated with initial systemic and intravitreal therapy: a 10-year retrospective cohort study

**DOI:** 10.1007/s10792-025-03518-1

**Published:** 2025-05-05

**Authors:** Daniel Ho Tak Wong, Jennifer Chien Hui Hung, Kenneth Kai Wang Li

**Affiliations:** 1 Department of Ophthalmology, United Christian Hospital, Hospital Authority, 130 Hip Wo Street, Kwun Tong, Hong Kong SAR, China; 2 Department of Ophthalmology, Tseung Kwan O Hospital, Hospital Authority, 2 Po Ning Lane, Tseung Kwan O, Hong Kong SAR, China; 3 Department of Ophthalmology, School of Clinical Medicine, LKS Faculty of Medicine, The University of Hong Kong, 21 Sassoon Road, Pokfulam, Hong Kong SAR, China

**Keywords:** Endogenous endophthalmitis, *Klebsiella* endophthalmitis, Intravitreal antibiotics

## Abstract

**Background:**

Management of endogenous endophthalmitis (EE) is challenging. *Klebsiella* EE, in particular, carries a guarded prognosis and poor visual outcome. We aimed to test the hypothesis that a certain group of *Klebsiella* EE would carry a better prognosis when presenting with specific signs, compared to the worse-performing group when treated with initial systemic and intravitreal therapy.

**Methods:**

A retrospective cohort study was conducted across two local tertiary hospitals to review all cases of *Klebsiella* EE from January 2013 to December 2022. Anatomical success was defined by retention of the globe, without intractable retinal detachment or phthisis bulbi. Functional success was defined as achieving a visual acuity of better than 1.3 logMAR.

**Results:**

A total of 56 proven cases of EE were identified. 31 subjects (55.4%) had *Klebsiella* EE. Of those, 31 eyes of 21 subjects met the inclusion criteria. 4 subjects were unconscious or cognitively impaired at initial presentation. The factors that predict a higher chance of successful treatment with systemic antibiotics and intravitreal antibiotics only, in the *Klebsiella* EE group, were a better visual acuity at presentation, lack of conjunctival injection, absence of corneal edema, hypopyon, panophthalmitis, and the presence of a fundal view (*p* < 0.02). The probability of overall treatment success was greater than 50% if the initial visual acuity was better than or close to finger counting at one meter (*p* = 0.006).

**Conclusion:**

Universal screening of patients with *Klebsiella* infection and the identification of earlier EE presentation with these prognostic factors that predict a positive or less severe outcome are crucial for the prompt initiation of treatment within this narrow therapeutic window.

## Introduction

### Challenge of management of Klebsiella endogenous endophthalmitis

Management of endogenous endophthalmitis (EE) is challenging. It requires prompt diagnosis, and liaising with medical internist. EE has a poor prognosis with 59–68% of patients having a final visual acuity of hand motion or worse [1, 2]. *Klebsiella pneumoniae*, with its hypervirulent strains, carries an even worse prognosis in EE [3]. It has been identified as a separate group of EE that is difficult to treat and specifically associated with poor visual outcomes [4]. Evisceration or enucleation have been frequently required [5–7].

### Lack of studies focusing on prognostic factors that predict a less severe disease

Due to the lack of clear guideline and uniform consensus, and the variation of causative organisms and their virulence internationally, it is important to identify the less severe and poor prognostic factors at the onset to formulate the best treatment plan for each case promptly. To the authors’ knowledge, there has not been any study identifying the factors that predict a positive or less severe outcome after an initial course of systemic and intravitreal antibiotics in particular in EE, with a strict definition of treatment success and failure, in either a single causative organism group or all pathogens collectively. Previous long-term studies focused on identifying the poor prognostic factors in *Klebsiella* EE that required evisceration, and the most recent definition of endophthalmitis treatment success and failure was not adopted [8, 9]. Danielescu et al.[10] defined anatomical success by retention of the globe, without intractable retinal detachment or pthisis bulbi, and defined functional success as achieving a visual acuity better than 20/400 (1.3 logMAR), following the World Health Organization 10th Edition of International Statistical Classification of Diseases and Related Health Problems (ICD-10) classification where 20/400 or worse is considered blindness[11]. With the implementation of timely screening of patients with *Klebsiella* septicaemia in the recent decade, milder cases of *Klebsiella* EE are identified earlier and can recover with earlier treatment.

### Existing treatment strategies of Klebsiella EE and their limitations

The treatment of *Klebsiella* EE involves systemic and local therapy. When blood culture results are still pending, empirical intravenous broad-spectrum antimicrobials are typically initiated by the internists as per microbiologists’ local guidelines and then adjusted according to sensitivities. To rapidly increase the antibiotic load in the vitreous and bypass the blood-retinal barrier, intravitreal antibiotic injections are usually given. Vitrectomy to obtain cultures, decrease viral load and improve anti-microbial agents penetration are performed selectively in cases, and less universally than post-op endophthalmitis, due to the lack of major controlled trials [4]. The vitrectomy rates in *Klebsiella* EE were reported to be very low, in 8.1% to 26.8% of cases [10], except one small case series that reported vitrectomies performed in 100% of their cases, which involved 6 eyes 4 patients [12]. Liaising with the parent medical team to eradicate the primary septic source, most commonly hepatobiliary infections which may require abscess drainage, is of utmost importance. Very severe cases without visual prognosis would require evisceration or enucleation.

There are more limitations and challenges in *Klebsiella* EE compared to more commonly seen post-op endophthalmitis. Patients can be systemically ill or even unconscious and intubated, with deranged liver and renal function, or in shock with disseminated intravascular coagulation [13, 19], rendering them too ill to tolerate further procedures and surgery. Patients’ family members have to be involved in those who lack the capacity to consent. Signs of early EE may not be obvious while the patient is admitted for workup for sepsis, and diagnosis of EE and identifying the source of infection is often delayed, which is even worse with highly virulent organisms.

### The screening and management of EE at our centers in the study period

Patients initially presenting mainly with ocular symptoms of EE would present to the accident and emergency departments at our hospitals and then seen by our ophthalmologists at the clinic. For patients with major systemic symptoms and signs, for example, pyrexia, abdominal pain, abnormal blood tests, would be admitted to the medical or surgical wards. If they presented with ocular signs during admission, or their blood or abscess cultures returned to be *Klebsiella pneumoniae* positive, consultation requests would be sent to our ophthalmology department via the electronic clinical medical system, and most of the patients would be attended by our ophthalmologists within 24 h. Detailed ophthalmic evaluations were feasible using mounted, portable slit lamps, indirect ophthalmoscopes and B-scan in clinic or intensive care settings.

In the study period, for patients with an initial suspicion of EE, tap and inject would be arranged as an emergency procedure within the same day. Aqueous and vitreous samples, if obtained, were sent to the microbiology department for culture and sensitivities. Injection of two intravitreal antibiotics, ceftazidime 2mg in 0.1 ml with either vancomycin 1 mg in 0.1 ml or amikacin 0.4 ml in 0.1 ml, would be given in the same setting. In general, intravitreal vancomycin and cefuroxime were in the study period our first line empirical antibiotics for EE of any cause, in line with general recommendations [2–4]. If it was a culture-confirmed *Klebsiella* EE, either intravitreal cefuroxime alone, or a combination of amikacin and cefuroxime would be administered. The patients would be reviewed daily, and subsequent doses of intravitreal antibiotics were usually given 48–72 h later if indicated. The choice of antibiotics could be altered depending on the culture sensitivities. Clinical course varied greatly depending on the pathogen and stage of disease at presentation. If the eye showed marked signs of improvement, no further injections would be given. In cases of painful blind eyes with no visual prognosis, rapid progression to panophthalmitis, and corneal perforation, evisceration would be performed. Panophthalmitis is defined as severe inflammation of all layers and structures of the eye, including the sclera, cornea, retina, choroid, vitreous, and the inflammation may extend to orbital tissues causing orbital cellulitis [14, 15]. For cases with visual potential, rescue vitrectomy would be performed on a case-by-case basis depending on the risk–benefit ratio and co-morbidities, as determined by the senior vitreoretinal specialists. The indications included patients with clinical deterioration after one to two intravitreal injections of antibiotics and those with a bilateral condition, for whom vitrectomy was performed on the worse eye to salvage some vision. Other factors to consider include fitness for surgery under general anesthesia and any systemic comorbidity that would preclude surgery.

## Subjects and methods

### Study setting and population

We aimed to test the hypothesis that a certain group of *Klebsiella* EE would carry a better prognosis when presenting with specific signs, compared to the worse-performing group, when treated with initial systemic and intravitreal therapy. This is a retrospective cohort study at the United Christian and Tseung Kwan O Hospitals in Hong Kong, China, with a catchment population of 1.2 million. A review of all the cases which were diagnosed with EE at these two hospitals was conducted. The electronic medical records were retrieved using the diagnosis coding of “endophthalmitis” within a 10-year period from January 2013 to December 2022 across all hospital wards. This study received approval from the local Research Ethics Committee of the Kowloon Central/ Kowloon East of the Hospital Authority, Hong Kong (Reference: KC/KE-23–0179/ER-3) and complied with the declaration of Helsinki.

### Inclusion and exclusion criteria

Inclusion criteria were (1) subjects diagnosed with EE with a positive systemic culture, (2) isolation of *Klebsiella pneumoniae*, (3) survival of more than three months after the initial diagnosis of endophthalmitis by an ophthalmologist, and (4) treatment with systemic antibiotics, with or without at least one intravitreal injection after the diagnosis.

Exclusion criteria were (1) non-infective uveitis, (2) recent ocular surgery or trauma in the 12 months before diagnosis of endophthalmitis, (3) exogenous causes of endophthalmitis (e.g. keratitis related or post-operative endophthalmitis).

### Outcomes measured

The primary outcome of this study was the treatment success, which included anatomical and functional success. The definitions of these were adopted from the review article published in 2020 by Danielescu et al.[10] Anatomical success was defined in most EE case series by retention of the globe, without intractable retinal detachment or pthisis bulbi [10]. Functional success was defined as achieving a visual acuity of better than 1.3 logMAR [10] and an eye without signs of inflammation at 3 months of follow up. The visual acuity was measured with Snellen chart, or a hand-held near chart for subjects examined on a stretcher, and was converted to logMAR for analysis.

Secondary outcomes included visual acuity change, number of additional surgery e.g. evisceration, number of injections, ocular symptoms and signs, the intravitreal antibiotics used, days after admission when being attended by an ophthalmologist, other comorbidities including malignancy, diabetes mellitus, intravenous drug use, systemic immunosuppressive therapy, endocarditis, acquired immune deficiency syndrome (AIDS), autoimmune disease, intensive care unit admissions, if any. The presence of fundal view is defined as, irrespective of the presence or absence of vitritis, the ability of the examiner to discern the optic nerve, macula and more than half of the peripheral retina at first visit.

The number of confirmed cases of EE in our hospitals per year and the percentage of EE in sepsis cases within our hospitals per year were also calculated, using the discharge summary diagnosis codes: “Sepsis”, “candida sepsis”, sepsis due to “staphylococcus, streptococcus, sepsis due to other gram- negative organisms” to identify the cases of sepsis. The number of *Klebsiella* septicaemia cases annually were identified with the diagnosis codes: “*Klebsiella* septicaemia” and “*Klebsiella pneumoniae* septicaemia”.

### Statistical analysis

Statistical analysis was performed using SPSS software (v26, IBM Corp. Armonk, NY, USA). Relationships between categorical variables were assessed using Chi-square test, and that between continuous data were assessed using t-test for parametric and Mann–Whitney U test for non-parametric data. The probability of treatment success was analysed with logistic regression. A p-value of less than 0.05 is considered significant.

## Results

### Population selection and proportion of Klebsiella EE cases

**Fig. 1:**
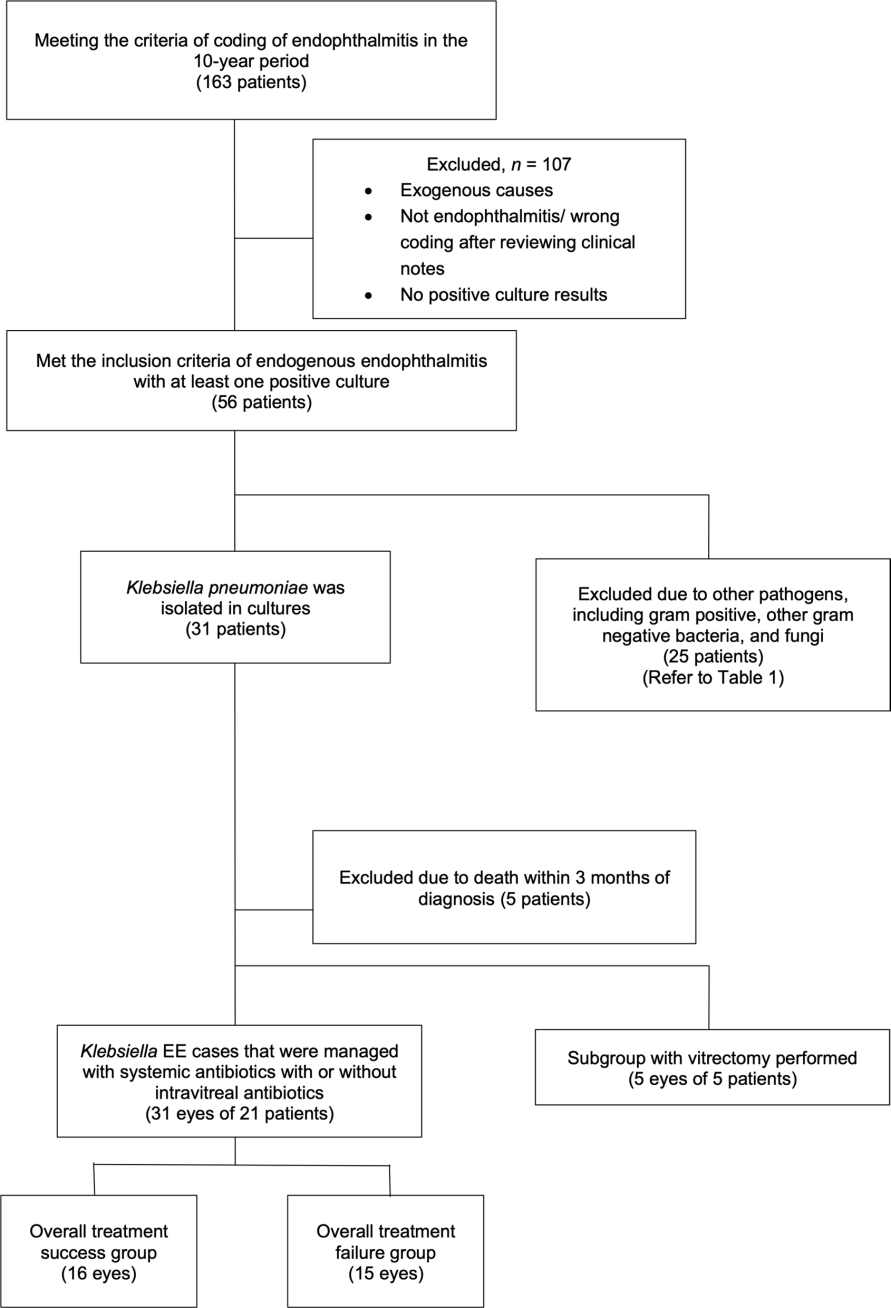
Flow diagram of the recruitment results of our study. EE: endogenous endophthalmitis

A flow diagram of the recruitment results is shown in Fig. 1. A total of 163 subjects who had a coding of endophthalmitis in the 10-year period were identified. After excluding the exogenous causes and incorrect coding, 56 cases were identified as EE with at least one positive culture. The microbial spectrum is detailed in Table 1. More than half (55.4%) (*n* = 31) of the EE cases were of *Klebsiella pneumoniae* origin. The number of confirmed cases of *Klebsiella* EE in our hospitals during the study period was 3.1 per year. The number of *Klebsiella pneumoniae* cases across the hospital sites annually was 260. This figure remained steady over the decade. The percentage of EE in *Klebsiella* sepsis cases per year was 0.12%. Among the *Klebsiella* EE, 5 subjects were excluded due to death within 3 months of diagnosis, and 5 eyes of 5 subjects received pars plana vitrectomy during the disease course. Of the 31 subjects, 21 (31 eyes) remained for analysis.

**Table 1:** Microbial spectrum of confirmed endogenous endophthalmitis cases

Species	Total subjects, *n*	Positivity rate, %
Gram-positive organisms	16	28.5
*Streptococcus anginosus*	1	
*Methicillin-resistant Staphylococcus aureus* (MRSA)	3	
*Staphylococcus aureus* (Methicillin sensitive)	6	
*Streptococcus agalactiae*	3	
*Streptococcus suis*	1	
*Streptococcus dysgalactiae*	2	
Gram negative organisms	34	60.7
*Klebsiella pneumoniae*	31	
*Klebsiella oxytoca*	1	
*Escherichia coli*	2	
*Citrobacter koseri*	1	
Fungus		14.2
*Candida albicans*	8	
Total subjects that met the inclusion criteria of endogenous endophthalmitis with at least one positive culture^	56	

^ some subjects have mixed growth. Among the *Klebsiella pneumoniae* positive subjects, two had mixed growth with *Candida*, one had mixed growth with MRSA

### Patient characteristics and ocular features

The subject characteristics of the *Klebsiella* EE cohort are outlined in Table 2. The mean age of our cohort was 67.2 ± 11.1, with 54.5% as male subjects. More than half of the subjects (*n* = 14, 63.6%) had diabetes mellitus, and two were diagnosed upon admission. Three subjects (13%) had an unremarkable past health history. Four subjects (18.2%) had a history of malignancy, with one undergoing chemotherapy for cholangiocarcinoma. None of the cases had a history of intravenous drug use. 16 cases (72.7%) had liver abscesses. 18 (81.8%) cases were consultations from the medical, surgical in-patient wards or the intensive care unit. Only 4 cases (18.1%) presented to our clinicians first with ocular symptoms, but all of them had associated symptoms of chills and rigors. The average number of days after admission before being attended by an ophthalmologist was 4.5 ± 8.8 (Ranging from 0 to 42).

**Table 2:** General characteristics of included cases of *Klebsiella* endogenous endophthalmitis

Patients	Number of patients	22
	Number of eyes	31
Age (years)	Min–max (median)	51–95 (64.5)
	Mean ± SD	67.2 ± 11.1
Gender, *n* (%)	Male	12 (54.5%)
	Female	10 (45.5%)
Laterality, *n* (%)	Left	4 (18.2%)
	Right	8 (36.3%)
	Bilateral	10 (45.5%)
Ethnicity, *n* (%)	Chinese	22 (100%)
Presentation, *n* (%)	Outpatient	1 (4.5%)
	Intensive care unit	3 (13.6%)
	Accident and emergency department	3 (13.6%)
	Medical or surgical in-patient wards	15 (68.2%)
Death from 3 months after diagnosis to 1 year after diagnosis, *n*		3 (13.6%)
For the first half of our cases (2013 – 2017), death from second to fifth year after diagnosis, *n*		0
Initial visual acuity of the affected eye (LogMAR)	Min–max (median)	0–3 (1.3)
	Mean ± SD	1.54 ± 1.05

SD: standard deviation

The average presenting visual acuity was 1.54 ± 1.05 logMAR, while the acuity of 6 eyes from 4 subjects could not be tested due to a lack of verbal response; these subjects were either intubated or were cognitively impaired. 12 eyes (38.7%) of 10 subjects had a presenting visual acuity of finger counting or worse, and two eyes (6.5%) of two subjects were already no light perception (NLP) at presentation. The common ocular complaints described were blurred vision, conjunctival hyperemia, periorbital swelling and erythema and an increase in floaters. Conjunctival injections were found in 15 (48%) of the involved eyes, while periorbital cellulitis, hypopyon and cornea edema were found in 5 (16.1%), 10 (32.3%), and 15 (48.3%) eyes at the initial assessment, respectively.

### Treatment and overall outcomes

The overall anatomical and functional success and failure rates are tabulated in Table 3. Overall treatment and functional success were achieved by 16 eyes (51.6%) of 9 subjects. Anatomical failure was seen in 13 eyes (41.9%) of 12 subjects. 8 eyes (25.8%) of 7 subjects were eviscerated, and including them, a total of 13 eyes (41.9%) of 11 subjects remained NLP. All 21 subjects (100%) received broad-spectrum intravenous antibiotics according to sensitivities, 23 eyes (74.2%) of 17 subjects (81.8%) received intravitreal antibiotics. All the *Klebsiella* species in our series were sensitive to ceftriaxone, imipenem and gentamicin. Ceftriaxone was the most commonly given antibiotic (to 12 subjects, 52.3%), followed by carbapenems (meropenem and ertapenem) to 9 subjects (42.8%). In addition, metronidazole and quinolone antibiotics (levofloxacin, ciprofloxacin) were used in 8 cases (38.1%) and 6 cases (28.6%), respectively. All subjects that received intravitreal injections had at least one of the injections as ceftazidime each time. 8 eyes (25.8%) of 6 subjects did not receive intravitreal antibiotics. Among them, three were painful eyes of two subjects who had NLP without fundal view at presentation and proceeded to evisceration. The other 5 eyes of 4 subjects had retinitis only with clear vitreous and hence were treated with systemic antibiotics only. 12 eyes (38.7%) of 8 subjects had a final visual acuity of better than or equal to 0.3 logMAR.

**Table 3:** Treatment success and failure rates of included eyes of *Klebsiella* endogenous endophthalmitis

*n* = 31 eyes	Success, *n* (%)	Failure, *n* (%)
Functional	16/31 (51.6%)	15/31 (48.4%)
Anatomical	18/31 (58.1%)	13/31 (41.9%)
Overall Treatment	16/31 (51.6%)	15/31 (48.4%)

### The overall treatment success subgroup

In the subgroup of overall treatment success with systemic and intravitreal antibiotic, the presenting visual acuity was 0.77 ± 0.71 logMAR, compared to 2.52 ± 0.48 logMAR in the overall treatment failure group (*p* < 0.001) (Table 4). The most commonly used systemic antibiotic, in combination or isolation, was ceftriaxone (8 subjects, 61.5%), metronidazole (6 subjects, 46.2%), carbapenems (4 subjects, 30.8%) and ciprofloxacin (3 subjects, 23.1%). All subjects that received intravitreal injections had at least one of the injections as ceftazidime each time. They received 1.38 ± 1.20 injections on average, not significantly different from 1.93 ± 1.39 injections in the treatment failure group (p = 0.24). 54.5% of the eyes had improvement seen within 72 h after first intravitreal injection if this was given, and 45.4% had static picture. 77.8% of the eyes had improvement in the retinitis and/or vitritis seen within 72 h of the second injection, if this was given. The average time taken for the resolution for all active fundal lesions and inflammation was 77.8 ± 52.0 days.

**Table 4:** Prognostic factors in selected *Klebsiella* endogenous endophthalmitis cases

		Overall Treatment failure group (*n* = 15)	Overall Treatment success group (*n* = 16)	*p*-value	Anatomical failure group (*n* = 13)	Anatomical success group (*n* = 18)	*p*-value
*Demographics*							
Age (Years)		69.00 ± 12.07	64.31 ± 7.80	0.21^a^	69.46 ± 12.95	64.50 ± 7.37	0.23^a^
Gender	Male	7 (46.67)	10 (62.50)	0.38^c^	5 (38.46)	12 (66.67)	0.12^c^
	Female	8 (53.33)	6 (37.50)		8 (61.54)	6 (33.33)	
Diabetes	Yes	10 (66.67)	10 (62.5)	0.81^c^	8 (61.54)	12 (66.67)	0.77^c^
	No	5 (33.33)	6 (37.5)		5 (38.46)	6 (33.33)	
HbA1c level (%)		7.63 ± 2.65	9.31 ± 3.18	0.15^a^	7.74 ± 2.82	8.96 ± 3.08	0.29^a^
Immunosuppression	Yes	1 (6.67)	1 (6.25)	1.00^c^	0 (0.0)	2 (11.11)	0.50^c^
	No	14 (93.33)	15 (93.75)		13 (100.0)	16 (88.89)	
Malignancy	Yes	2 (13.33)	4 (25.00)	0.65^c^	1 (7.69)	5 (27.78)	0.36^c^
	No	13 (86.67)	12 (75.00)		12 (92.3)	13 (72.22)	
Time when first attended by ophthalmologist after admission (Days)		3.00 ± 3.84	6.19 ± 9.85	0.12^b^	3.08 ± 4.09	5.78 ± 9.34	0.14^b^
Intensive care admission	Yes	2 (13.33)	3 (18.75)	1.00^c^	1 (7.69)	4 (22.22)	0.37^c^
	No	13 (86.67)	13 (81.25)		12 (92.31)	14 (77.78)	
*Blood tests*							
Deranged liver function	Yes	12 (80.00)	13 (81.25)	1.00^c^	10 (76.92)	15 (83.33)	0.68^c^
	No	3 (20.00)	3 (18.75)		3 (23.08)	3 (16.67)	
Presence of liver abscess	Yes	14 (93.33)	10 (62.50)	0.08^c^	12 (92.31)	12 (66.67)	0.19^c^
	No	1 (6.67)	6 (37.50)		1 (7.69)	6 (33.33)	
Impaired renal function	Yes	3 (20.00)	1 (6.25)	0.33^c^	3 (23.08)	1 (5.56)	0.28^c^
	No	12 (80.00)	15 (93.75)		10 (76.92)	17 (94.44)	
White cell count (× 10^9^/litre)		17.03 ± 4.10	18.82 ± 6.75	0.38^a^	17.16 ± 4.41	18.52 ± 6.40	0.52^a^
C-reactive protein (milligrams/ litre)		168.23 ± 85.44	188.25 ± 96.66	0.59^a^	158.55 ± 83.89	193.00 ± 94.13	0.35^a^
*Presentation*							
Presenting visual acuity (logMAR)		2.52 ± 0.48	0.77 ± 0.71	< 0.001^b^*	2.48 ± 0.52	1.02 ± 0.93	< 0.001^b^*
Presenting intraocular pressure (mmHg)		19.91 ± 11.48	16.33 ± 3.43	0.36^b^	19.78 ± 12.81	16.82 ± 3.54	0.83^b^
Conjunctival injection	Yes	15 (100.00)	0 (0.00)	< 0.001^c^*	13 (100.00)	2 (11.11)	< 0.001^c^*
	No	0 (0.00)	16 (100.00)		0 (0.00)	16 (88.89)	
Cornea edema	Yes	15 (100.00)	0 (0.00)	< 0.001^c^*	13 (100.00)	2 (11.11)	< 0.001^c^*
	No	0 (0.00)	16 (100.00)		0 (0.00)	16 (88.89)	
Panophthalmitis	Yes	5 (33.33)	0 (0.00)	0.02^c^*	5 (38.46)	0 (0.00)	0.01^c^*
	No	10 (66.67)	16 (100.00)		8 (61.54)	18 (100.00)	
Hypopyon	Yes	10 (66.67)	0 (0.00)	< 0.001^c^*	10 (76.92)	0 (0.00)	< 0.001^c^*
	No	5 (33.33)	16 (100.00)		3 (23.08)	18 (100.00)	
Fundal view	Yes	3 (20.00)	14 (87.50)	< 0.001^c^*	3 (23.08)	14 (77.78)	< 0.001^c^*
	No	12 (80.00)	2 (12.50)		10 (76.92)	4 (22.22)	
*Treatment regimen*							
Number of intravitreal antibiotics injections		1.93 ± 1.39	1.38 ± 1.20	0.24^a^	1.62 ± 1.19	1.67 ± 1.41	0.92^a^

^*^p < 0.05, ^a^t-test, ^b^Mann-Whitney U test, ^c^Chi square test

### Comparing the main systemic and intravitreal antibiotic group with vitrectomy subgroup of Klebsiella EE

After excluding cases who died during admission, only 5 eyes of 5 subjects with *Klebsiella* EE underwent vitrectomy during the period. One was intubated at presentation, and the rest had 1.0logMAR, finger counting (FC), hand movement (HM) and light perception (LP) vision respectively. One case received pars plana vitrectomy (PPV) on the same day of diagnosis, and the rest had PPV after one to two intravitreal injections. All of them received further one to two intravitreal antibiotics injections after the PPV. Four of the cases ended up with no light perception (NLP), three had pthisical changes and one was eviscerated. The remaining case had a vision of 2.1logMAR. None of the cases achieved functional or anatomical success.

### Prognostic factors analysis

The results and comparison of the baseline demographics, initial blood test results, and prognostic factors between the two groups of overall success and failure are summarised in Table 4. In the *Klebsiella* EE group, better visual acuity at presentation, lack of conjunctival injection, absence of corneal edema, hypopyon, panophthalmitis, and presence of a fundal view were associated with higher treatment success rates (*p* < 0.02). By logistic regression analysis, the probability of overall treatment success was greater than 50% when the initial visual acuity was better than logMAR 1.85 (*p* = 0.006). Similarly, the presence of conjunctival injection, corneal edema, periorbital cellulitis, hypopyon, and a lack of fundal view at presentation, would predict treatment failure. Other systemic factors, including white cell count, C-reactive protein and liver function, as well as the presence of liver abscess, malignancy and diabetes, were not found to be statistically significant in our analysis. The presentation with unilateral or bilateral disease does not statistically significantly predict the treatment outcome in our cohort (*p* = 0.38).

## Discussion

EE is not as commonly seen as post-operative endophthalmitis, with an estimated proportion of only 2 to 8% of all cases. [2] In one study in Western Australia, the average frequency of EE was only 1.6 per 1 million population per year [16]. The incidence of EE is higher in our locality, with an incidence of 5.6 cases per million population per year, partially attributed to the endemic *Klebsiella* infection, as liver abscess is the most common extraocular focus of infection in EE in Asia [17, 18]. Infection occurs when the colonizing *Klebsiella* pneumoniae is able to invade through the barriers of the gastrointestinal tract predominantly, or the respiratory tract [19, 30]. EE caused by *Klebsiella* continues to account for more than half of our EE cases in our center, and the incidence has remained stable across the last two decades. [19]

Eviscerations were performed in up to 41% of *Klebsiella* EE cases [4, 9, 20], and 25.8% of our eyes involved. This is because in *Klebsiella* EE, extensive subretinal abscesses tend to develop early, leading to the destruction of retinal tissue and loss of central visual acuity. [5, 21] The presence of hypopyon and unilateral involvement in *Klebsiella* infection may also herald a poorer prognosis. [4, 9].

As identified by the review article from Danielescu et al., for anatomical success, most authors of case series between 2011 and 2020 reported a rate of 64.3 to 100% for all cases of EE, and a large range from 25 to 83.3% for *Klebsiella* EE [10]. For functional success, most authors reported a rate of 4.5 to 64% for all causes of EE, and from 16.6 to 50% of the eyes with *Klebsiella* EE.[10]. The highest success rate (50%) was in a small case series where all eyes underwent vitrectomy, but it only included 6 eyes of 4 patients [12]. Our functional success rate is around the higher end of the range reported by Danielescu. We postulate that this may be due to the inclusion of milder cases in our study population due to the universal screening protocol.

With an increased awareness of the importance of universal screening of endophthalmitis in *Klebsiella* septicaemia among clinicians, early referrals for ophthalmic screening have increased in the recent decade locally. Our study has highlighted the factors that predict a higher chance of successful treatment with systemic antibiotics and intravitreal antibiotics only in the earlier stages of disease, in the *Klebsiella* EE subgroup. These factors include the lack of conjunctival injection, absence of cornea edema, panophthalmitis, and hypopyon, and the presence of fundal view. These factors imply an earlier presentation of *Klebsiella* EE, and for this reason, outcomes are better. A visual acuity of better than logMAR 1.85 (which was close to finger counting at 1 m, i.e. 1/60 as defined in ICD-10 [11]) at presentation carried a better prognosis, although all other clinical signs need to be taken into account. We postulate that it is important to identify the cases during the initial stages when the bacteria had not, or just breached the blood retinal barrier, and milder and earlier stages of diseases would be easier and possible to treat to retain good vision. Although diabetes mellitus is known to be a frequent association with *Klebsiella* EE, [3, 22] our study did not find a difference of prevalence of diabetes between the success and failure groups.

For mild cases exhibiting the identified favourable or less severe prognostic factors, we recommend initiating collaboration with internists and microbiologists for systemic antibiotics. In cases with vitreous involvement, treatment should involve at least one intravitreal antibiotic injection (which includes a third-generation cephalosporin, e.g. ceftazidime), administered 48–72 h apart, to *Klebsiella* EE cases, in accordance with local antimicrobial guidelines. Close observation of the patient's condition, monitoring for improvement or worsening, is crucial before deciding to proceed to further injections and vitrectomy. The decision should take into account the patient’s prognosis, general co-morbidities, and fitness for surgery and anaesthesia. For patients with unfavourable factors, it is essential to inform the patients and relatives about the aggressiveness of the pathogen and the poor prognosis. Additionally, they should be cautioned about the possibility of evisceration or enucleation.

Our study only included a very small subgroup that received vitrectomy. The role of early vitrectomy in EE has been widely debated due to a lack of guidelines [5]. For all causes of EE in general, obtaining vitreous samples with vitrectomy is superior to traditional tapping procedures for obtaining positive cultures because the volume obtained is larger. It also reduces the chance of tractional breaks during the blind tapping procedure. Vitreous biopsy with vitrectomy was also superior in endophthalmitis etiology compared to blood cultures. [17, 21, 23, 24] In terms of treatment, vitrectomy helps to remove inflammatory material and the ocular septic foci. It also helps increase the antimicrobial diffusion in the vitreous chamber. [25–27] However, for *Klebsiella* EE in particular, it can be technically challenging and risky, due to the poor view of the posterior segment and aggressiveness of the disease. Dense retinal abscess, vitreous pus and coagulum in advanced *Klebsiella* EE can be difficult to remove intraoperatively, and lensectomy and retinectomy may be required. There is also a significant post-op retinal redetachment rate or re-operation rate [28, 29]. It may be inoperable and a futile effort in cases with advanced disease. In larger studies in Asia, vitrectomy was not found to improve the visual outcome of *Klebsiella* EE cases [8, 9], with final visual outcome worse than FC in 80% of the early operated cases in one study [8]. We believe that in *Klebsiella* EE, vitrectomy can be considered for cases in the middle of the severity spectrum with careful case selection, after a comprehensive analysis of the risk–benefit ratio for surgery and anesthesia, and the co-morbidities.

Our study is limited by its moderate sample size for *Klebsiella* EE, considering EE is still an uncommon entity. Each eye from a bilateral involvement subject was analysed separately, which could lead to overrepresentation. However, none of the systemic parameters was found to be statistically significant for our conclusions. We also did not investigate into other treatment modalities, including intravitreal steroid. Exclusion of the group which received vitrectomy could potentially result in bias. Future large trials can be conducted to look into the favourable prognostic factors of patients who will achieve treatment success from vitrectomy, and the best timing of medical and surgical interventions for all pathogens, including *Klebsiella*. This data would be invaluable in the clinical decision-making process.

## Conclusion

Systemic and intravitreal antibiotics are more likely to be successful in *Klebsiella* EE that is less severe. The factors that predict a higher chance of successful treatment with systemic antibiotics and intravitreal antibiotics only, in *Klebsiella* EE, were a better visual acuity at presentation, lack of conjunctival injection, absence of cornea edema, panophthalmitis, hypopyon, and the presence of fundal view. A visual acuity that is better than or close to finger counting at one meter at presentation is indicative of a more favorable prognosis. Hence, universal screening of patients with *Klebsiella* septicaemia and the identification of these less severe prognostic factors are crucial for the prompt initiation of treatment within this narrow therapeutic window. The results of our study can provide the evidence to justify clinicians’ clinical decision. Conversely, the presence of more severe signs may indicate that conservative treatment could be insufficient, prompting clinicians to consider and discuss more proactive and invasive treatments and the prognosis with patients and family early in the clinical decision process. This prognostic information is important in the counselling process for the patient and their relatives, especially when the patient is septic and, during difficult times, when the patient is unable to give informed consent for treatment.

## Data Availability

The data that support the findings of this study are available from the corresponding author upon reasonable request.
